# Set1 Targets Genes with Essential Identity and Tumor-Suppressing Functions in Planarian Stem Cells

**DOI:** 10.3390/genes12081182

**Published:** 2021-07-29

**Authors:** Prince Verma, Court K. M. Waterbury, Elizabeth M. Duncan

**Affiliations:** Department of Biology, University of Kentucky, Lexington, KY 40506, USA; prince.verma@uky.edu (P.V.); ckwa224@uky.edu (C.K.M.W.)

**Keywords:** planarian, tumor suppressor, histone methylation, Set1

## Abstract

Tumor suppressor genes (TSGs) are essential for normal cellular function in multicellular organisms, but many TSGs and tumor-suppressing mechanisms remain unknown. Planarian flatworms exhibit particularly robust tumor suppression, yet the specific mechanisms underlying this trait remain unclear. Here, we analyze histone H3 lysine 4 trimethylation (H3K4me3) signal across the planarian genome to determine if the broad H3K4me3 chromatin signature that marks essential cell identity genes and TSGs in mammalian cells is conserved in this valuable model of in vivo stem cell function. We find that this signature is indeed conserved on the planarian genome and that the lysine methyltransferase Set1 is largely responsible for creating it at both cell identity and putative TSG loci. In addition, we show that depletion of *set1* in planarians induces stem cell phenotypes that suggest loss of TSG function, including hyperproliferation and an abnormal DNA damage response (DDR). Importantly, this work establishes that Set1 targets specific gene loci in planarian stem cells and marks them with a conserved chromatin signature. Moreover, our data strongly suggest that Set1 activity at these genes has important functional consequences both during normal homeostasis and in response to genotoxic stress.

## 1. Introduction

Cells have powerful abilities to mitigate cancer-causing events, such as abnormal growth and DNA replication errors. The genes and mechanisms underlying these functions are essential for maintaining the structural and functional integrity of complex tissues, particularly those that undergo constant cell turnover such as the skin and intestines. Unsurprisingly, mutation or misregulation of genes that confer these gate-keeping abilities, i.e., tumor suppressor genes (TSGs), predisposes cells to malignant transformation. There is thus a critical need to uncover and dissect all cellular mechanisms of tumor suppression, including those regulating DNA repair, genome surveillance, and antiviral responses, in order to identify potential drug targets and strategies that could restore tumor suppressor function in cancer cells. Although DNA sequencing of human cancer cells has identified many potential TSGs and mouse models have validated some, these systems have significant practical limitations that prevent discovery and high-throughput screening in vivo, particularly for TSGs operating at the earliest stages of tumorigenesis. 

Planarians are free-living flatworms best known for their remarkable regenerative capacity. They also have several unique, yet relevant, biological traits that make them a powerful model of in vivo stem cell biology. Adult planarians maintain a large, heterogeneous pool of multi and pluripotent stem cells that endow them with two extraordinary abilities: (1) to maintain indefinite cell turnover and homeostasis and (2) to regenerate entirely new animals from nearly any small piece of amputated tissue. Strikingly, despite maintaining this large population of highly plastic, proliferating cells, planarians do not normally exhibit visible signs of cancer. Moreover, these animals have the capacity to recover from high doses of genotoxic stress, for example as induced by ionizing radiation [1]. These data strongly suggest that planarians possess robust mechanisms to prevent, monitor, and repair genome instability and point to their untapped potential as a model for discovering new, fundamental mechanisms of tumor suppression. At the same time, perturbation of some conserved tumor suppressors [2,3,4] and fundamental tumor-suppressing functions [5] has been shown to cause tumor-like outgrowths in planarians, revealing that planarian tumorigenesis shares physiological features with that of humans.

At a molecular level, many fundamental proteins and pathways identified in planarians are highly conserved in domain structure and function with their human homologs, including those that regulate genome stability and gene expression [6,7,8,9]. For example, we have previously characterized the planarian homologs of Set1 and MLL1/2, enzymes that catalyze histone H3K4me3 at the promoters of active genes, and shown that the planarian enzymes are highly conserved in both their chromatin-modifying activity and the relationship of this activity to gene expression [6]. Further, we showed that Set1 targets genes required for planarian stem cell function and, interestingly, that it marks them with a distinct chromatin signature, i.e., unusually wide patterns of H3K4me3 that extend across gene bodies. Notably, a separate study simultaneously identified this same chromatin signature, at TSGs in normal human cells and that it is dramatically diminished at these loci in many cancer cells [10]. Given that planarian stem cells are the only dividing cells in these animals and must carefully regulate proliferation and genome integrity to prevent tumorigenesis during both homeostasis and regeneration, we hypothesized that this conserved chromatin feature likely marks genes required for planarian tumor suppression as it does in humans.

Here, we show that the wide H3K4me3 peak signature is indeed conserved in marking specific types of genes of the planarian genome and that, moreover, this gene-specific pattern is largely catalyzed by Set1 in planarian stem cells. In addition, we show that animals depleted of the Set1 enzyme display phenotypes consistent with loss of tumor suppressor gene function, including an inability to respond appropriately to a clinical dose of ionizing radiation. Together, these data strongly suggest that planarians are an excellent model in which to uncover novel TSGs, dissect TSG mechanisms, and study TSG function in vivo. They also point to potentially exciting connections between transcriptional regulation, cellular niches, and DNA damage response (DDR) pathways, paving the way for many future studies.

## 2. Materials and Methods

### 2.1. Mnase ChIP-seq

Planarian stem cells were isolated by FACS from untreated adult *S. mediterranea* animals as previously described [11,12]. Briefly, animals were dissociated into a single cell suspension in calcium and magnesium-free buffer containing 0.5% BSA (CMFB), stained for one hour in Hoechst 33342, and processed on a BD Influx Cell Sorter. 800 K cells were collected from the X1 gate and mixed with 9.2 M Drosophila S2 cells. The X1 + S2 cell pool was then used to perform Mnase ChIP-seq as previously described [13,14] with an antibody to H3K4me3 (Millipore 07-473). The lysis buffer used for isolation of nuclei was modified to 10mM sodium chloride (NaCl) and 10mM potassium chloride (KCl) to accommodate the lower osmolarity of planarian cells compared to mammalian cells [15]. Mnase digestion was optimized to obtain mononucleosomes, which were then used for ChIP. ChIP-seq libraries were then generated from H3K4me3 precipitated DNA and DNA from input chromatin using an optimized protocol with reagents from the Illumina TruSeq kit (RS-122-2001, Illumina, Inc., San Diego, CA, USA).

### 2.2. Analysis of ChIP-seq Datasets

Previously published ChIP-seq reads [6] and Mnase-ChIP-seq reads were aligned to the Rink lab v4 version *S. mediterranea* genome [16] using bowtie2 [17]. Peaks were called and their widths (lengths) determined by MACS2 [18].Differences in H3K4me3 between control (*unc22(RNAi)*) and knockdown (*set1(RNAi)*) were determined using diffReps [19]. Peaks were mapped to gene models using BEDtools [20] and all further quantitative analyses were carried out using R [21] and figures produced using ggplot2 [22].

### 2.3. Planarian Culture, RNAi and Radiation

An asexual strain (CIW4) of *Schmidtea mediterranea* worms were maintained in 1X Monjüic water with gentamicin supplemented at 50ug/mL as previously described [23]. RNAi was performed as previously described [24]. Briefly, dsRNA was transcribed in vitro from cloned constructs in the pPR-T4P vector using RNA T7 polymerase, mixed with cow liver paste, and fed to animals every 3–4 days. Radiation was done with an X-Rad 225XL Precision X-ray irradiator.

### 2.4. Whole-Mount In Situ Hybridization and Immunofluorescence

*Piwi*-1 and histone H3 S10 phosphorylation (H3S10ph) detection was performed as previously described [25,26,27]. Briefly, riboprobes were made by in vitro transcription with T7 polymerase from the pPR-T4P vector and used in Whole-mount in situ hybridization (WISH). Worms were killed in 5% NAC, fixed in 4% formaldehyde, then dehydrated in methanol and stored at −20 °C. Worms were then rehydrated, bleached with 5% formamide/1.2% H_2_O_2_/0.5XSSC for 1.5 h, treated with 2μg/mL ProteinaseK, and post-fixed with 4% formaldehyde before adding appropriate riboprobes and hybridizing overnight at 56 °C. Excess riboprobe was washed with multiple washes of increasing stringency (2XSSC to 0.2XSSC, all with 0.1% Tween-20) and incubated overnight at 4 °C with α-DIG-POD antibody. Antibody was washed with 0.5%TritonX100/1XPBS before developing with a tyramide-conjugated fluorophore diluted in 0.006% H_2_O_2_/borate buffer. Immunofluorescence for H3S10ph was performed after WISH by incubating with an antibody to H3S10+T11 (ab32107) at 1:1000 for 48 h at 4 °C. Primary antibody was washed with 0.5%TritonX100/1XPBS before adding either anti-rabbit HRP or anti-rabbit-Alexa 555 overnight at 4 °C. Animals in which HRP secondary was used were then developed with tyramide-conjugated fluorophore as with fluorescent WISH. Hoechst 33342 was added during the last wash. Worms were then mounted in 30% glycerol/4M urea/0.1% TritonX-100/2.5% DABCO and imaged on a Leica SP8 DLS confocal using 10× and 63× objectives.

## 3. Results

### 3.1. A Broad Histone H3K4me3 Chromatin Signature Is Conserved in Planarians

H3K4me3 has long been known to mark transcription start sites (TSSs) and correlate positively with gene expression [14,28]. Although H3K4me3 is typically observed to mark TSSs in fairly sharp peaks and increase in peak height with increased gene expression, recent papers have shown that the width of H3K4me3 peaks also correlates significantly with gene expression levels and serves as a signature of genes with particular functions [10,29,30,31]. Specifically, broad H3K4me3 peaks have been shown to mark both genes that confer cell-specific identity [10,29] and those with tumor-suppressing function [10]. Our previous work revealed that broad H3K4me3 peaks are similarly locus specific in planarian stem cells; the planarian homologs of H3K4me3 KMTases Set1 and MLL1/2 target distinct genomic loci, with Set1 catalyzing much broader peaks than MLL1/2 [6]. We therefore asked if broad H3K4me3 is also a signature of cell identity and TSGs in planarians. 

**Figure 1:**
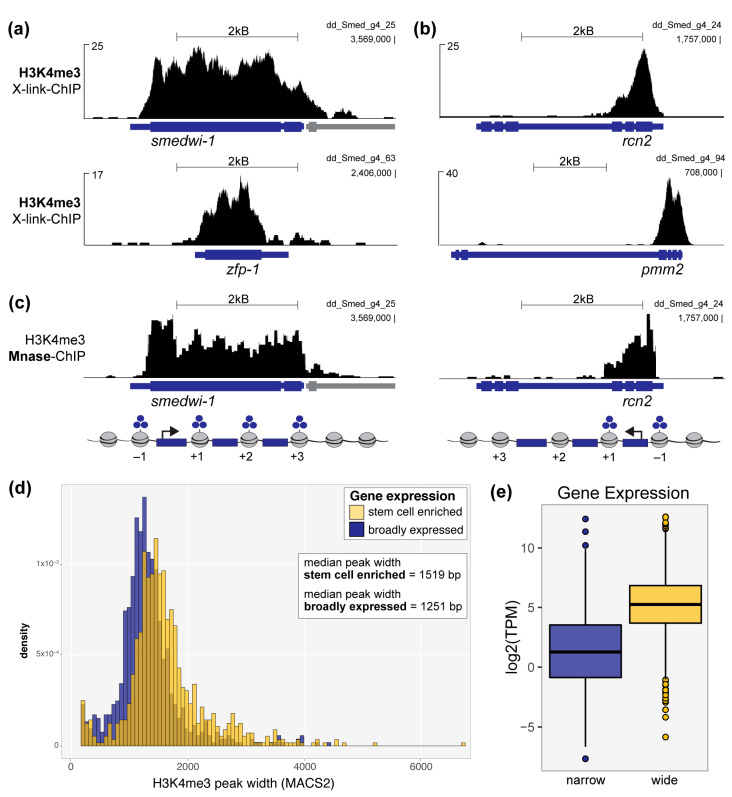
Wide H3K4me3 peaks mark identity genes in planarian stem cells. H3K4me3 signal at the genomic loci of: (**a**) *smedwi-1* and *zfp-1*, two well-established identity genes for planarian stem cells, and (**b**) *rcn2* and *pmm2*, genes that are robustly but not specifically expressed in planrian stem cells. Data in (**a**), (**b**) and (**d**) are from a ChIP-seq experiment in which input chromatin was formadlehyde crosslinked and mechanically sheared (X-link-ChIP). (**c**) H3K4me3 signal from an independent ChIP-seq experiment in which input chromatin was enzymatically fragmented into mononucleosomes using micococcal nuclease (Mnase-ChIP; see also Supplemental Figure S1); (**d**) Plot comparing the widths of H3K4me3 peaks called (MACS2) at the promoters of genes preferentially expressed in planarian stem cells (blue) versus those at broadly expressed genes (yellow). H3K4me3 peaks at stem cell genes are significantly wider, on average, than peaks at genes with broader expression patterns, *p* < 2.2 × 10^−16^ (Welch two-sample t-test). (**e**) Plot showing the average expression of genes marked by wide H3K4me3 peaks (blue) versus narrow peaks (yellow); “wide”= top 10% widest H3K4me3 MACS2-called peaks, “narrow”= bottom 10% narrowest H3K4me3 MACS2-called peaks; the expression difference between genes marked by wide versus narrow peaks is signifcant, *p* < 2.2 × 10^−16^ (Welch two-sample t-test).

To address this question, we first aligned our previously published H3K4me3 ChIP-seq data from wild-type planarian stem cells [6] to the newest version of the *S. mediterranea* genome [16]. As this assembly is significantly less fragmented and redundant than the previous version, it facilitates more accurate and complete alignments of genomic data. Indeed, when we looked at genomic loci that encode well-established planarian stem cell genes, such as *smedwi-1* and *zfp-1* (Figure 1a), we observed broad peaks of H3K4me3 that extended across their entire gene-coding regions. Notably, this feature is not solely the result of an overall increase in H3K4me3 signal, we also observed many genes marked by H3K4me3 peaks with equally high or taller summits that were not correspondingly wide (Figure 1b). In addition, we note that these peaks are not only significantly wide based on their extension across a planarian gene coding region, but also in comparison to “wide” H3K4me3 peaks on the human genome. A previous report defined wide H3K4me3 peaks as >4 kB at human gene loci [10]. As the average planarian gene is significantly smaller than the average human genes (approximately 4x smaller by comparing average sizes reported for planarian [32] and human [33] genes), a H3k4me3 peak on the planarian genome may be considered wide at significantly <4 kB.

We then asked whether the apparent increase in peak width was due to a tendency for chromatin fragments of increased shearing size to immunoprecipitate with the H3K4me3 antibody or if the nucleosomes positioned across these genes were in fact all trimethylated. To address this issue, we performed H3K4me3 ChIP-seq with mononucleosome fragments that we generated by micrococcal nuclease (Mnase) digest (Figure S1). Mnase-ChIP allows for high-resolution mapping of individual nucleosomes, compared with the imprecise and varied fragment sizes generated by formaldehyde cross-linking (X-linking) and mechanical shearing (Figure S1a,b) [34]. Our high-resolution ChIP-seq data confirmed that the broad H3K4me3 peaks seen at planarian stem cell identity genes are indeed a reflection of their methylation status across each gene locus at the +1, +2, etc. nucleosomes (Figure 1c). Using MACS2 to call H3K4me3 peaks across the genome with both X-linked and Mnase datasets, we further found that these two ChIP-seq methods show similar distributions of H3K4me3 peak width (Figure S1c). 

To query the correlation between broad H3K4me3 signal and cell identity genes at a genome-wide scale, we compared H3K4me3 peaks called at the promoters of genes with robust and enriched expression in the stem cell population (or X1 population; [6]) to those found at the promoters of genes with broader expression patterns. Notably, all genes we included in the “broadly expressed” comparison group did have a MACS2-identified peak at its genome locus and RNA-seq detected expression in stem cells. Nevertheless, genes with enriched expression in the stem cell population are marked with significantly wider H3K4me3 peaks than those genes with broader expression patterns (Figure 1d). We then asked if H3K4me3 peak width correlates positively with gene expression, as seen in other models [10,30]. We compared genes with the widest H3K4me3 peaks (top 10% MACS2-called peak widths) to those marked by the narrowest H3K4me3 peaks (bottom 10% MACS2-called peak widths) and found that genes marked by wide H3K4me3 peaks are expressed at significantly higher levels than those marked by narrow H3K4me3 peaks (Figure 1e) in planarian stem cells. Together, these data support the hypothesis that the wide H3K4me3 chromatin signature is conserved at planarian stem cell identity genes.

### 3.2. Set1 Targets TSGs in Planarian Stem Cells

**Figure 2:**
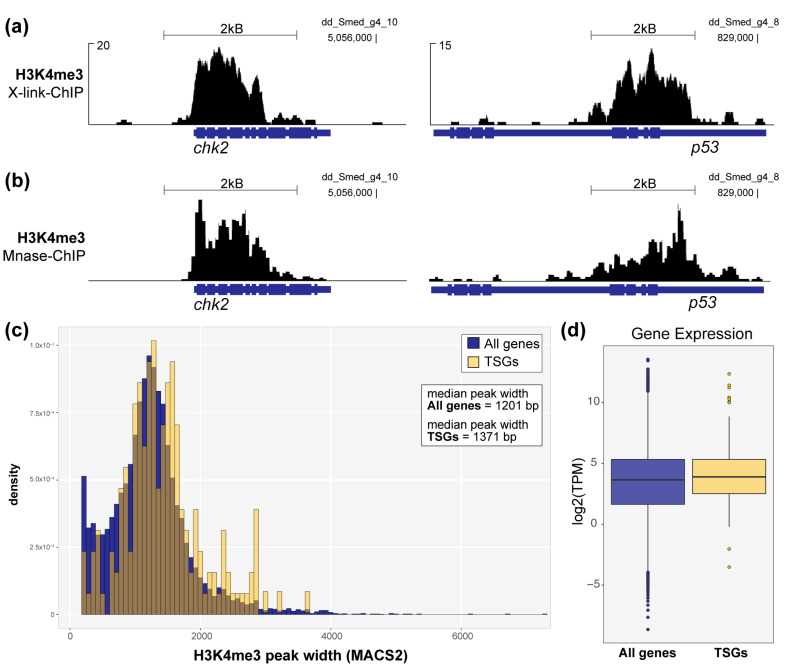
Wide H3K4me3 peaks mark the genomic loci of TSG homologs in planarian stem cells. (**a**) H3K4me3 signal from X-link-ChIP-seq and (**b**) Mnase-ChIP-seq at the loci of two TSG homologs, *chk2* and *p53*. (**c**) Plot comparing the width distributions of H3K4me3 peaks at TSG promoters (yellow) versus all genes marked by H3K4me3 at their promoters in planarian stem cells (blue). The difference in H3K4me3 peak width is small but significant, *p* < 2.2 × 10^−16^ (Welch two-sample t-test; X-link-ChIP-seq data). (**d**) Plot showing average expression of TSG homologs (yellow) versus all H3K4me3-marked genes in planarian stem cells.

After establishing that the broad H3K4me3 signature is conserved in marking identity genes in planarian stem cells, we then asked if this signature is also conserved in marking TSGs, as shown in humans [10]. We found multiple examples of planarian TSG homologs that are decorated with wider than average H3K4me3 peaks in planarian stem cells (Figure 2a) and confirmed this finding in H3K4me3-Mnase-ChIP (Figure 2b). To address this link in a non-targeted way, we then compared the widths of H3K4me3 peaks at the promoters of all identifiable planarian TSG homologs with all H3K4me3 promoter peaks found in planarian stem cells. Importantly, because there are too few functionally-defined TSGs in the planarian literature for a statistically meaningful genomic analysis, we exclusively define “planarian TSG homologs” as genes with annotations [35] that match a publicly available and highly curated list of established TSGs [36]. Although this analysis is constrained by the fact that there are many human TSGs without clear planarian homologs and that there are likely many unidentified and uncharacterized TSGs that are essential to the robust tumor-suppressing abilities observed in planarians, our analysis did find that the promoters of planarian TSGs homologs are often marked by moderately, but significantly, wider H3K4me3 peaks (Figure 2c). However, these TSG homologs do not show significantly higher expression than other H3K4me3-marked genes (Figure 2d). We note that TSGs may be marked by this chromatin signature in homeostatic planarian stem cells but only expressed as needed, e.g., in response to genotoxic stress.

We previously reported that Set1 is the lysine methyltransferase that catalyzes wide H3K4me3 peaks at specific genomic loci in planarian stem cells [6]. We then wanted to address whether Set1 specifically targets TSGs in planarians. To start, we aligned the previously published H3K4me3 ChIP-seq data from control and *set1(RNAi)* stem cells [6] to the new *S. mediterranea* genome [16], identified loci at which H3K4me3 changed significantly using diffReps [19], and mapped all differential H3K4me3 peaks to a new set of corresponding gene models [35]. Because our previous analysis showed that Set1 creates wide H3K4me3 peaks, we performed additional analyses to determine whether changing the default diffReps widow size (1000 bp) would improve our ability to identify additional Set1 targets, particularly those with the widest H3K4me3 peaks. Indeed, we found that both decreasing and increasing the window size allows diffReps to identify new differential H3K4me3 loci and that increasing the window size identifies potential Set1 target genes with increased H3K4me3 width (Supplemental Figure S2). However, our analyses also found that increasing the window size beyond 1500 bp added significant numbers of false positive target genes, due to both mapping errors (Supplemental Figure S3c) and the additive effects of neighboring peaks (Supplemental Figure S3d). We therefore combined the genes identified using 500 bp, 1000 bp, and 1500 bp diffReps windows to create a complete Set1 target gene list (Supplemental Table S1).

**Figure 3:**
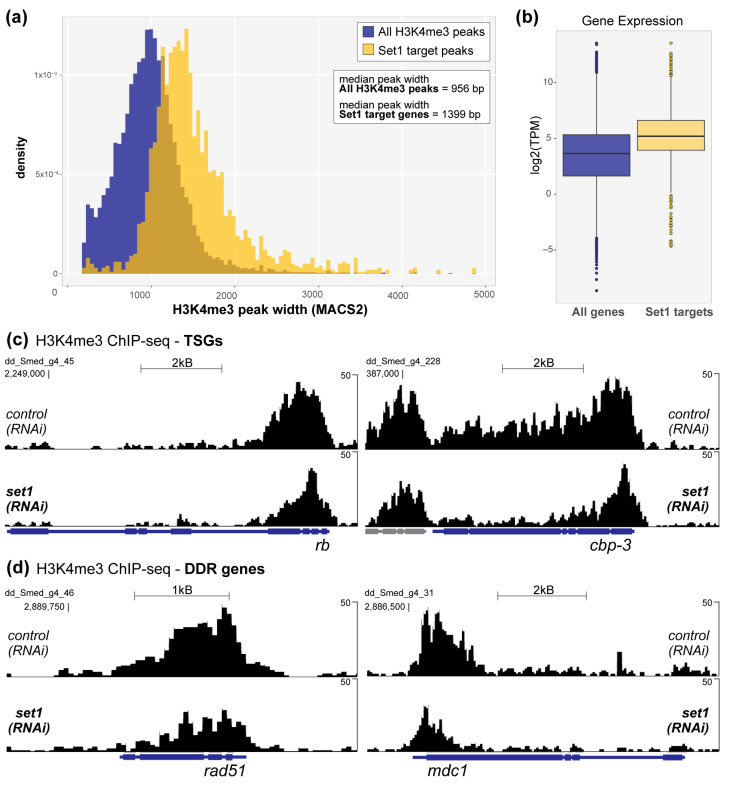
Set1 catalyzes wide H3K4me3 peaks at target gene promoters in planarian stem cells. (**a**) Plot of peak width distributions for all H3K4me3 peaks called at gene promoters (blue) and Set1 target gene promoters (yellow) in stem cell ChIP-seq. H3K4me3 peaks at Set1 target genes are significantly wider, on average, than other H3K4me3 peaks, 2.2 × 10^−16^ (Welch two-sample t-test). (**b**) Plot of average expression of all genes marked by a H3K4me3 peak at the promoter (blue) versus those targeted by Set1 (yellow); Set1 target gene expression is significantly higher, *p* = 8.3 × 10^−7^ (Welch two-sample t-test). (**c**) H3K4me3 ChIP-seq signal from both control and *set1(RNAi)* stem cells at the loci of two TSG homologs, *rb* and *cbp-3*. H3K4me3 is significantly reduced in *set1(RNAi)* stem cells at both promoters: log2FC= −0.97 (*p*-adj= 2.3 × 10^−5^; diffReps) at *rb*, and log2FC= −1.08 (p-adj= 7.8 × 10^−10^; diffReps) at *cbp-3*. (**d**) H3K4me3 is also significantly reduced in *set1(RNAi)* stem cells at many DNA damage response (DDR) gene loci, including *rad51* (log2FC= −0.75, p-adj= 4.4 × 10^−4^; diffReps) and *mdc1* (log2FC= −1.04 log2FC, p-adj= 5.5 × 10^−6^; diffReps). All ChIP-seq data are from X-linked ChIP.

As expected, we observed that Set1 target genes are marked by significantly wider H3K4me3 peaks at their promoters as compared to all H3K4me3 promoter peaks (Figure 3a). Planarian Set1 target genes are also more highly expressed than genes with non-Set1-dependent H3K4me3 (Figure 3b), a finding that was in keeping with both our previous analysis [6] and the increased expression of wide H3K4me3-marked human genes [10]. Moreover, this expanded list of Set1 target genes in planarian stem cells includes many homologs of well-known human TSGs, including that of Rb and CBP/p300 (Figure 3c). In addition, many planarian Set1 targets are homologs of DDR pathway genes (Figure 3d).

### 3.3. Set1 Is Required for Tumor-Suppressing Functions in Planarians

**Figure 4:**
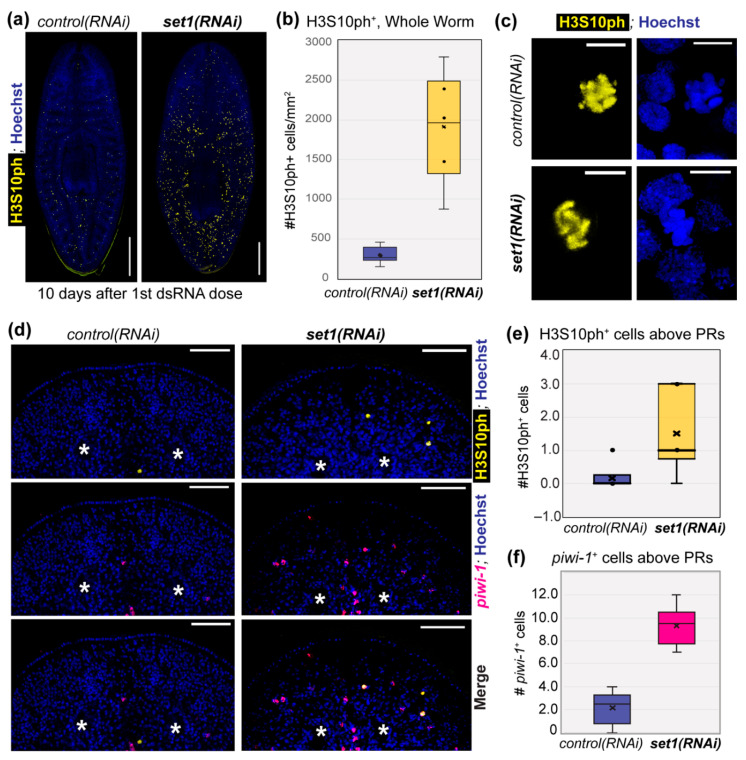
*set1(RNAi)* induces hyperproliferation and abnormal localization in planarian stem cells. (**a**) Representative images showing whole-mount immunofluorescent staining for histone H3 serine 10 phosphorylation (H3S10ph) in *control* and *set1(RNAi)* animals, 10 days after RNAi induction; scale bars = 500 µm. (**b**) Quantification of H3S10ph+ cells for all animals in the experiment shown in (**a**) (*n* = 6; *p* < 0.002; Student’s t-test). (**c**) Higher-magnification images of cells from the worms in (**a**); scale bars = 10 µm. (**d**) Higher-magnification images of worms from (**a**) showing the anterior region; photoreceptors (PRs) are marked with * and scale bars = 100 µm. (**e**) Quantification of H3S10ph+ (*p* < 0.05; Student’s t-test) and (**f**) piwi-1+ cells (*p* < 0.001; Student’s t-test) observed above the PRs in all worms per RNAi condition.

The correlation between Set1 activity, wide H3K4me3, and putative TSG loci prompted us to reexamine the phenotype of *(set1)RNAi* worms. A separate study found that the number of mitotic stem cells detected by histone H3 serine 10 phosphorylation (H3S10ph) was significantly increased at an intermediate time point after *(set1)RNAi* [7], an effect seen in many cell types and organisms upon loss of TSG expression or function [37,38,39]. We also observed a significant increase in histone H3S10ph^+^ cells 10 days after feeding planarians with dsRNA for *Smed-set1* (Figure 4a,b). To confirm that this increase in H3S10ph^+^ cells indicated an increase in mitotic cells, we looked at single H3S10ph^+^ cells with higher magnification (Figure 4c) and verified that these cells contained condensed chromosomes. Further, we not only observed an increase in H3S10ph^+^ cells across *set1(RNAi)* animals, but also noticed ectopic H3S10ph^+^ cells in regions of the animal where they are not normally found (e.g., anterior to the photoreceptors; Figure 4d). Our quantification of ectopic H3S10ph^+^ cells found this observation to be marginally significant, likely because only a subset of asynchronously dividing planarian stem cells are H3S10ph^+^. We therefore also quantified piwi-1^+^ cells anterior to the photoreceptors (Figure 4f) and found that *set1(RNAi)* animals have a significant number of ectopic piwi-1^+^ cells in this region. It is unclear why stem cells are present in abnormal locations in *set1(RNAi)* animals, but this phenotype is consistent with known effects of TSG loss, such as abnormal cell migration and/or invasion [40,41,42].

**Figure 5:**
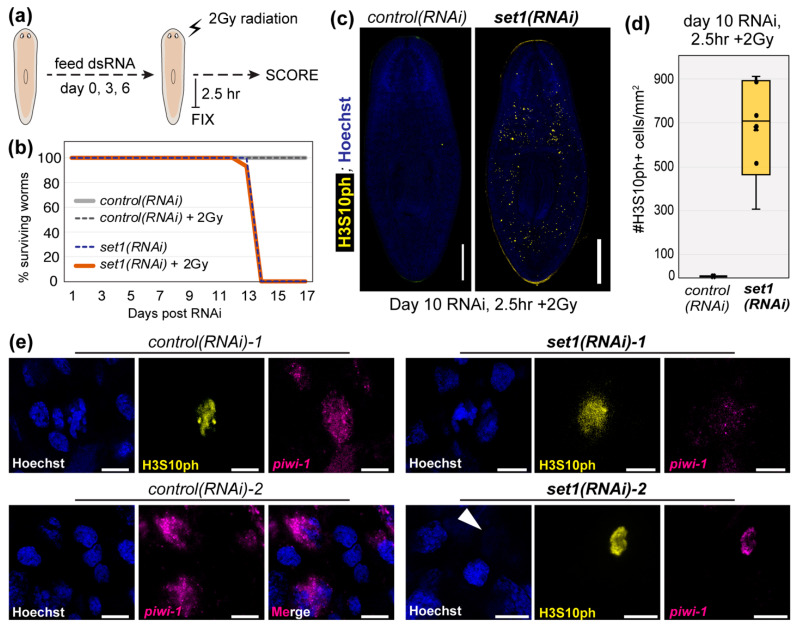
Planarian stem cells respond abnormally to ionizing radiation after set1(RNAi) depletion. (**a**) Schematic of the experimental setup for assessing the functional requirement of Set1 in the planarian response to a clinical fraction (2 Gy) of ionizing radiation. (**b**) Plot showing the survival curves of *control(RNAi)* and *set1(RNAi)* animals after 2 Gy radiation. (**c**) Representative images of H3S10ph in *control(RNAi)* and *set1(RNAi)* animals 2.5 h after 2 Gy radiation (10 days post-RNAi; scale bars = 500 µm. (**d**) Quantification of H3S10ph+ cells for all animals in the experiment shown in (**a**–**c**) (*n* = 6; *p* < 0.009; Student’s t-test). (**e**) Higher-magnification images of cells from the worms in (**c**); scale bars = 10 µm.

Our previous study reported that *set1(RNAi)* worms have increased sensitivity to the genotoxic stress induced by ionizing radiation [6]. However, that experiment used 1250 rads (12.5 Gy) of ionizing radiation, a dose known to induce apoptosis in the large majority of planarian stem cells with only a small subset surviving [1,43]. Given that our analyses found that planarian Set1 targets many genes in DDR pathways, wanted to know if depletion of Set1 impairs planarian stem cell recovery from DNA damage, such as that induced by ionizing radiation. We hypothesized that a sublethal, yet significant, dose of radiation would uncover Set1-dependent cellular functions but still preserve our ability to measure the cellular and molecular response of *control(RNAi)* stem cells to radiation treatment. We therefore fed adult planarian animals with dsRNA for a control gene or *Smed-set1* before exposing them to 2 Gy ionizing radiation, a dose that normally should not induce wide-scale apoptosis and is a common clinical fraction used during cancer treatment (Figure 5a). After monitoring these animals for two weeks, we observed that (1) both non-radiated and 2 Gy-radiated *control(RNAi)* worms appeared normal and (2) both non-radiated and 2 Gy-radiated *set1(RNAi)* animals showed head regression (HR) and ventral curling (VC) phenotypes and ultimately died (Figure 5b). However, 2 Gy radiation did not significantly impact the overall survival curve of *set1(RNAi)* animals, suggesting that it did not significantly accelerate cell death.

Because many chromatin-based responses to radiation are reported to occur within hours of exposure, we fixed both *control(RNAi)* and *set1(RNAi)* animals 2.5 h after 2 Gy radiation treatment and stained the proliferating stem cell population with both a piwi-1 riboprobe and the H3S10ph antibody. We detected very few H3S10ph^+^ cells in *control(RNAi)* animals at 2.5 h post-2 Gy radiation (Figure 5c,d), a finding consistent with the halt of cell cycle progression after DNA damage. Notably, both H3S10 phosphorylation [44] and H3T11 phosphorylation [45] have been observed to decrease significantly after ionizing radiation in mammalian cells, suggesting that planarian stem cells may respond to this genotoxic stress through a conserved mechanism. In contrast, we still observed many H3S10ph^+^ cells in *set1(RNAi)* animals at 2.5 h post-2 Gy radiation (Figure 5c,d), although considerably fewer than the average number detected in their non-irradiated counterparts (Figure 4a,b). We looked at the remaining H3S10ph^+^ stem cells in these worms at higher magnification to see if they had normal nuclear morphology. On the contrary, although the few remaining H3S10ph^+^ cells in *control(RNAi)* animals appeared normal (Figure 5e, “*control(RNAi)-1*”), the H3S10ph^+^ cells remaining in 2 Gy treated *set1(RNAi)* animals exhibited unusual features, including diffuse H3S10ph staining (Figure 5e, “*set1(RNAi)-1*”) and the absence of DNA staining (Figure 5e, “*set1(RNAi)-2*”). To address whether these findings were an artifact of signal amplification of H3S10ph, we repeated this experiment independently, stained this new set of RNAi +/- 2 Gy worms with the same H3S10ph antibody, and used an Alexa-555-conjugated secondary antibody to detect H3S10ph^+^ stem cells. As shown in Supplemental Figure S3, we observed comparable cells in this independent experiment. Although we do not yet understand what is causing these abnormal observations, we do not believe they are technical artifacts as we observe many neighboring cells with normal appearance. Together, these data support the hypothesis that 2 Gy radiation induces a cellular and molecular response in planarian stem cells and that this response is significantly altered in *set1(RNAi)* animals.

## 4. Discussion

Tumor-suppressing activities, including the regulation of cell proliferation, DNA damage surveillance and repair, and the maintenance of genome stability, are essential functions in multicellular organisms. Impaired tumor suppression not only renders cells more likely to acquire mutations that drive primary tumor formation, but also produces greater mutational heterogeneity within tumors, increasing the likelihood that some cells will become therapy resistant and metastatic and endanger the entire organism. At the same time, the genes driving these activities and functions are challenging to identify and study in vivo, in part due to the heterogeneous nature of cancer.

The planarian stem cell population bears many similarities to tumors: its cells are proliferative, heterogeneous, and can migrate. Moreover, some planarian stem cells can survive high doses of genotoxic stress (such as that delivered by cancer therapies like radiation and chemotherapies) and repopulate a new population of stem cells. At the same time, planarians normally do not exhibit many outward signs of tumorigenesis, suggesting that their stem cells have robust mechanisms for regulating these features that are reminiscent of cancer stem cells. Nevertheless, the specific details of these mechanisms and the molecules that drive them are largely unknown. In addition, although many apparently conserved TSG homologs have been shown to share some functions with their human counterparts, in many cases the functional conservation is unclear. For example, although RNAi targeting the *S.med* homologs of p53 or PTEN, both well-established human TSGs, each cause a burst of hyperproliferation at intermediate time points (much like RNAi of *set1*) they are also essential for stem cell differentiation and organism survival [2,3]. At the same time, many human TSGs are known to have essential functions in embryogenesis [46,47,48], indicating a conserved functional relationship between tumor-suppressing and stem cell functions. As many known TSGs are master regulators of gene expression or signaling, identifying the downstream targets of these proteins in vivo will likely provide valuable insight into the exact mechanisms controlling these functions.

Here, we show that specific loci in the planarian genome are marked by a conserved signature of tumor suppression, wide H3K4me3 peaks. Moreover, unlike in mammalian cells, it is clear the Set1 enzyme is largely responsible for this wide H3K4me3 signature in planarian stem cells, providing a useful way to identify new TSG candidates for in vivo screening. In addition to providing two distinguishing features of potential planarian TSGs, wide H3K4me3 and Set1 dependence, our findings also strongly suggest that there must be a molecular mechanism driving the establishment of this wide H3K4me3 signature at specific gene loci. In future studies, we aim to characterize this mechanism in order to uncover how the Set1 complex targets specific loci, particularly including TSGs. Such studies are likely to be more challenging in mammalian cells, where redundancy between an increased number of H3K4me3 methyltransferases makes it difficult to distinguish the enzyme responsible for the wide H3K4me3 signature.

The data presented here also uncover a strong connection between Set1 activity and the cellular response to DNA damage. Not only does planarian Set1 specifically target many genes known to function in this response, such as rad51, but also we report that *set1(RNAi)* causes stem cells to respond abnormally to DNA damage: many of these cells inappropriately maintain the H3S10ph mitotic mark (whereas stem cells in control animals do not, as seen in mammalian cells in response to radiation) and the majority of these H3S10ph^+^ cells exhibit disrupted nuclear morphology. These abnormalities suggest significant genomic instability, which may be the result of increased cell proliferation in the absence of TSG function. In future studies examining the mechanisms underlying Set1 function and those of its genomic targets, we will aim to understand how loss of Set1 and/or expression of its target genes causes this phenotype. The response of *set1(RNAi)* stem cells to radiation is also notably heterogeneous, an aspect of the phenotype that will be important to dissect and may reflect differences in cell cycle state, metabolic state, and/or differentiation potential.

It is also important to acknowledge that the Set1 enzyme, although best known as a histone-modifying enzyme, also has other known functions in cell biology. In particular, many relevant studies in yeast have demonstrated that Set1 has relevant roles in cell cycle progression and spindle assembly, most notably in response to DNA damage [44,45,46]. We will be following up on the potential connections between these findings and our own observations in planarian stem cells, keeping in mind that there are many key differences in the mechanism of both chromatin regulation and cell division between these species.

The retention of abnormal H3S10ph^+^ cells in *set1(RNAi)* + 2 Gy radiated animals is also intriguing in light of a recent study that showed how planarian stem cells alter their response to radiation in the context of injury [27]. This supports the possibility that stem cells in *set1(RNAi)* animals are not only responding differently to radiation damage, but also receiving different signals from their environment. By examining both the stem cell autonomous and non-autonomous effects of Set1 depletion, and that of its functional targets, we have the potential to gain insight into cancer stem cells and their metastasis. 

## Data Availability

Genomic data from this study are available from the Gene Expression Omnibus (GEO) database with accession numbers GSE73027 (RNA-seq from WT planarian stem cells), GSE74054 (all X-linked H3K4me3 ChIP-seq), and GSE180591 (Mnase H3K4me3 ChIP-seq).

## Supplementary Materials

The following are available online at https://www.mdpi.com/article/10.3390/genes12081182/s1, Figure S1: The H3K4me3 peak width distribution is highly similar between different methods of chromatin solubilization for ChIP-seq. Figure S2: Varying the diffReps window size parameter affects its ability to detect significant changes in H3K4me3 after *set1* depletion at some gene loci. Figure S3: A 2 Gy radiation treatment causes abnormal effects in *set1(RNAi)* stem cells. Table S1: Set1 target gene list.
