# Supplementary material for: Set1 Targets Genes with Essential Identity and Tumor-Suppressing Functions in Planarian Stem Cells
Source: Genes (Basel). 2021 Jul 29;12(8):1182. doi: 10.3390/genes12081182 (PMC8393678; doi:10.3390/genes12081182)
Supplement: Supplementary file 1 [file genes-12-01182-s001.zip › genes-1278917-supplementary/Supplemental figures S1-S3.pdf]

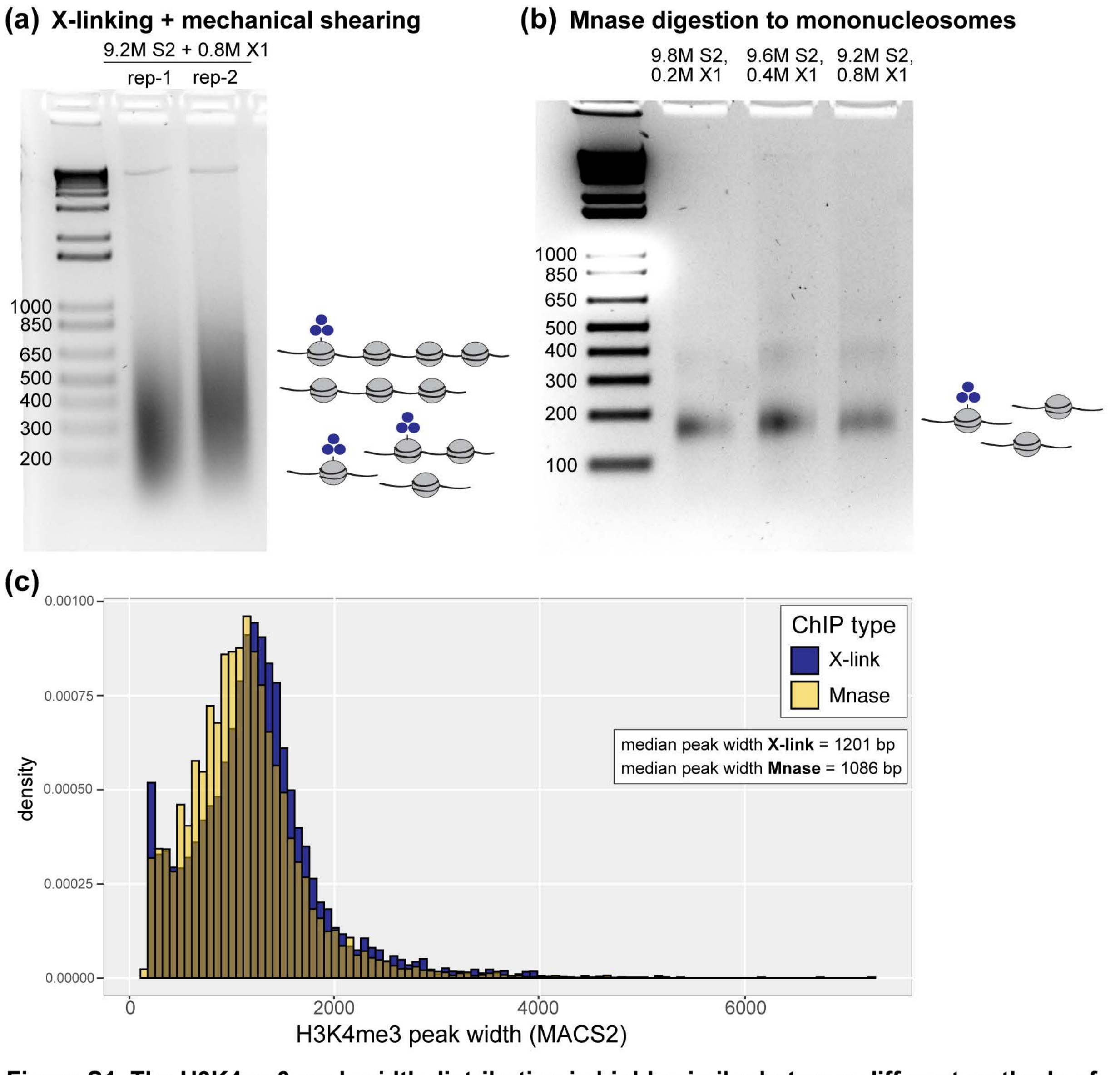

Figure S1. The H3K4me3 peak width distribution is highly similar between different methods of chromatin solubilization for ChIP-seq. Agarose gels showing the fragment size of DNA purified from input chromatin after (a) formaldehyde cross-linking and mechanical shearing (Covaris) and (b) Mnase digestion. In each case, Drosophila S2 cells were combined with FACS isolated X1 planarian stem cells in the numbers and ratios indicated. (c) Histogram of the distribution of H3K4me3 peak widths (MACS2 determined) found at gene promoters (within 600bp of a gene TSS) in both X-linked and Mnase generated ChIP-seq datasets; the two distributions show a small but significant difference, $p < 2.2e-16$.

(a) H3K4me3 changes in *set1(RNAi)* stem cells

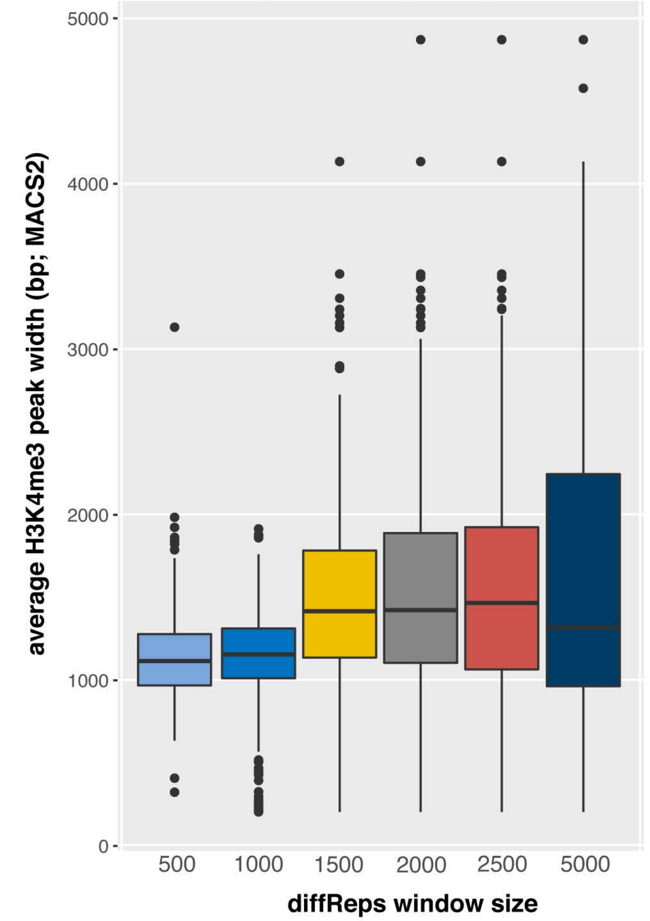

(b)

diffReps window size	# of differential windows added (compared to 1000bp)	mapping to >1 gene (comp. to 1000bp)	% windows added that are multi-mapping
500	97	0	0
1000	(1707)	(132)	(7.7)
1500	348	186	53.4
2000	410	329	80.2
2500	441	453	102.7
5000	228	328	143.9

(c)

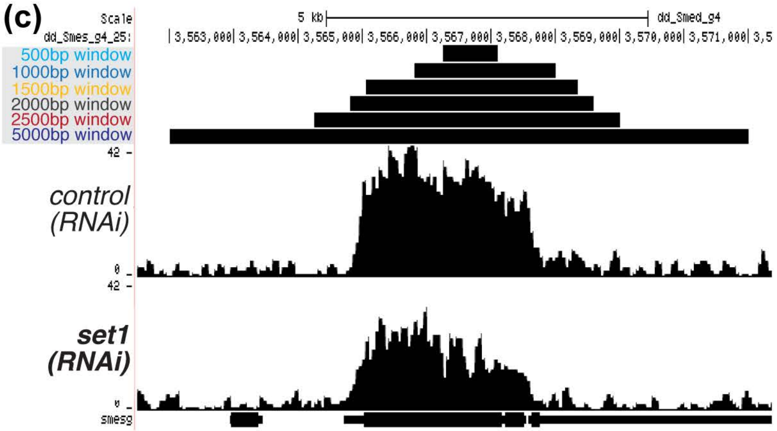

(d)

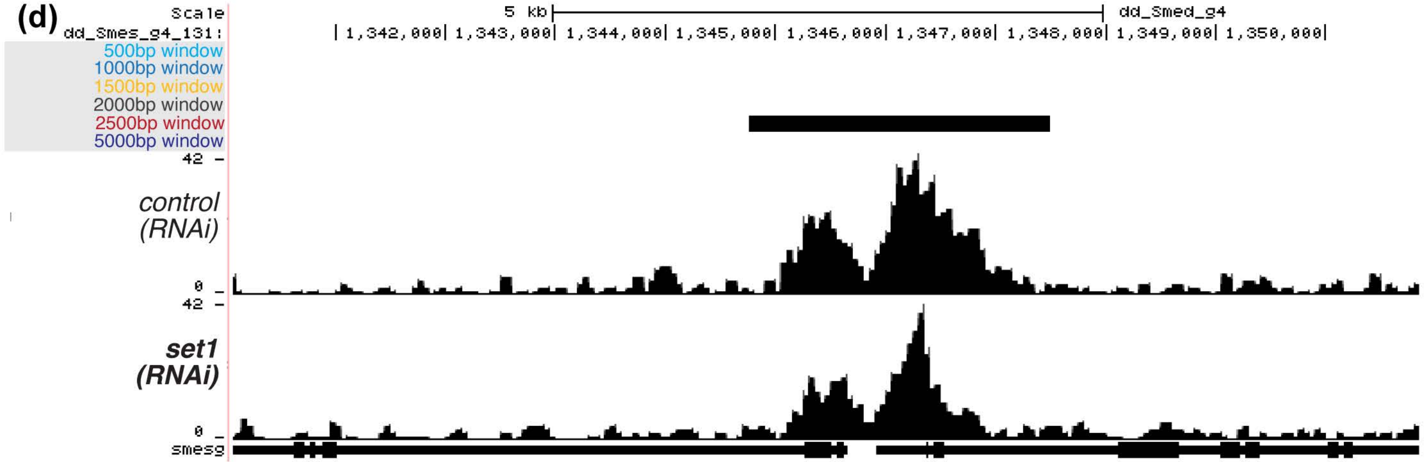

Figure S2. Varying the diffReps window size parameter affects its ability to detect significant changes in H3K4me3 after *set1* depletion at some gene loci. (a) Plot showing how changing the window size parameter in the diffReps analysis tool affects the average peak width (determined by MACS2) of those genes with differential H3K4me3 in *set1(RNAi)* stem cells versus *control(RNAi)* stem cells. (b) Table comparing the number of differential windows added per diffReps window size and number of added windows that map to more than one gene. (c) H3K4me3 ChIP-seq track of the *piwi1* locus and (d) at a locus which the 2500 diffReps window size misidentifies a Set1 target gene.

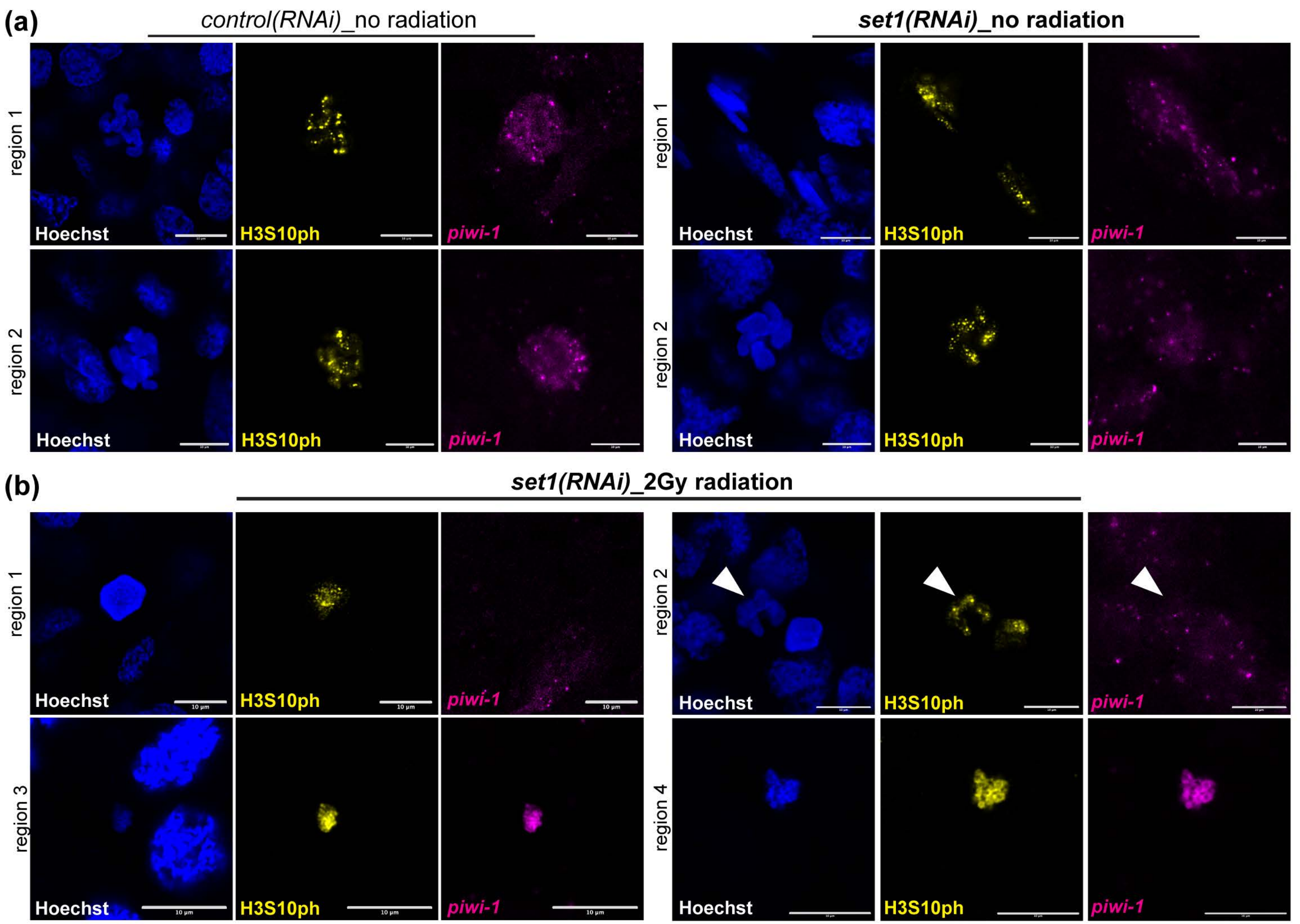

Figure S3. 2Gy radiation treatment causes abnormal effects in *set1(RNAi)* stem cells. (a) Representaive confocal images at 63X magnification in *control(RNAi)* and *set1(RNAi)* planarians from an independent experiment of that shown in Figure 5. In this experiment, H3S10ph antibody was detected with a secondary antibody conugated to Alexa-555 (*i.e.*, without amplification). In non-radiated *control(RNAi)* and *set1(RNAi)* animals, all cells analyzed appeared as shown with clearly condensed chromosomes that were marked by H3S10ph and diffuse *piwi-1* riboprobe signal. (b) Representative 63X magnification confocal images in *set1(RNAi)* planarians from this independent experiment, from worms fixed 2.5 hrs after 2Gy radiation treatment. Although nearly all cells in *control(RNAi)* worms were H3S10ph negative at 2.5h post 2Gy radiation, *set1(RNAi)* animals retained a signifcant number of cells that appear H3S10ph. Many of these H3S10ph^{+} cells display abnormal morphologies, although some do appear largely comparable to non-radiated cells (*e.g.*, the cell indicated by the white arrow in region 2). However, others show DNA that is condensed but not in the shape of chromosomes and colocalizing strongly with both H3S10ph and *piwi-1* signal. Moreover, as indicated by the scale bar, these highly condensed DNA structures are smaller than other Hoechst stained nuclei.
